# Acute Metabolic Stress Induces Lymphatic Dysfunction Through K_ATP_ Channel Activation

**DOI:** 10.1093/function/zqae033

**Published:** 2024-07-29

**Authors:** Hae Jin Kim, Charles E Norton, Scott D Zawieja, Jorge A Castorena-Gonzalez, Michael J Davis

**Affiliations:** Department of Medical Pharmacology & Physiology, University of Missouri, Columbia, MO 65212, USA; Department of Medical Pharmacology & Physiology, University of Missouri, Columbia, MO 65212, USA; Department of Medical Pharmacology & Physiology, University of Missouri, Columbia, MO 65212, USA; Department of Pharmacology, Tulane University School of Medicine, New Orleans, LA 70112, USA; Department of Medical Pharmacology & Physiology, University of Missouri, Columbia, MO 65212, USA

**Keywords:** contractile dysfunction, lymph pump, metabolic syndrome, reactive oxygen species, action potential, mitochondrial electron transport chain

## Abstract

Lymphatic dysfunction is an underlying component of multiple metabolic diseases, including diabetes, obesity, and metabolic syndrome. We investigated the roles of K_ATP_ channels in lymphatic contractile dysfunction in response to acute metabolic stress induced by inhibition of the mitochondrial electron transport chain. Ex vivo popliteal lymphatic vessels from mice were exposed to the electron transport chain inhibitors antimycin A and rotenone, or the oxidative phosphorylation inhibitor/protonophore, CCCP. Each inhibitor led to a significant reduction in the frequency of spontaneous lymphatic contractions and calculated pump flow, without a significant change in contraction amplitude. Contraction frequency was restored by the K_ATP_ channel inhibitor, glibenclamide. Lymphatic vessels from mice with global Kir6.1 deficiency or expressing a smooth muscle-specific dominant negative Kir6.1 channel were resistant to inhibition. Antimycin A inhibited the spontaneous action potentials generated in lymphatic muscle and this effect was reversed by glibenclamide, confirming the role of K_ATP_ channels. Antimycin A, but not rotenone or CCCP, increased dihydrorhodamine fluorescence in lymphatic muscle, indicating ROS production. Pretreatment with tiron or catalase prevented the effect of antimycin A on wild-type lymphatic vessels, consistent with its action being mediated by ROS. Our results support the conclusion that K_ATP_ channels in lymphatic muscle can be directly activated by reduced mitochondrial ATP production or ROS generation, consequent to acute metabolic stress, leading to contractile dysfunction through inhibition of the ionic pacemaker controlling spontaneous lymphatic contractions. We propose that a similar activation of K_ATP_ channels contributes to lymphatic dysfunction in metabolic disease.

## Graphical Abstract

**Graphical Abstract:**
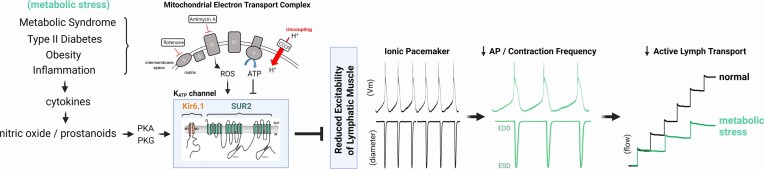
Depiction of events by which metabolic stress associated with multiple diseases/conditions of metabolic dysfunction results in ROS production and reduced ATP/ADP following inhibition of the mitochondrial electron transport complex, activating K_ATP_ channels in lymphatic muscle cells to inhibit the frequency of spontaneous action potentials generated and impair active lymph transport. For context, other pathways are shown that similarly modulate lymphatic muscle K_ATP_ channels, including Gain-of-Function (GoF) K_ATP_ channels expressed in patients with Cantu syndrome and the phosphorylation of K_ATP_ channels subsequent to PKA/PKG activation by nitric oxide/prostanoids.

Significance Statement Increasing evidence suggests that lymphatic contractile dysfunction contributes to the pathologies associated with multiple chronic diseases, including aging, obesity, type 2 diabetes, and metabolic syndrome, all of which are associated with metabolic stress. Although K_ATP_ channel activation plays a protective role in arterial smooth muscle under ischemic conditions, activation of K_ATP_ channels in lymphatic muscle decreases lymphatic muscle excitability and suppresses the generation of spontaneous contractions that aid in lymph propulsion. We investigated the possible contribution of K_ATP_ channels to the lymphatic contractile dysfunction associated with metabolic stress. Acute metabolic stress, induced by inhibition of the mitochondrial electron transport chain, led to increased K_ATP_ channel activity in lymphatic muscle, either through a reduction in the intracellular ATP/ADP ratio or increased production of ROS, resulting in impairment of the ionic pacemaker driving spontaneous contractions and active lymph transport. These actions were reversed by the K_ATP_ channel inhibitor, glibenclamide. Transgenic mice lacking functional K_ATP_ channels in lymphatic muscle were resistant to the effects of acute metabolic stress. Our findings point to a common role for K_ATP_ channels in the impaired lymphatic function observed in a number of metabolic diseases.

## Introduction

The lymphatic system plays a major role in tissue fluid homeostasis through the removal of excess fluid and protein filtered from blood capillaries into the interstitium.^1^ The accumulation of excess interstitial fluid, most often in dependent extremities, leads to the chronic condition lymphedema. Lymphedema may result from genetic mutations in critical genes controlling lymphatic vessel or valve development,^2^ surgical disruption of lymphatic networks,^3^ altered permeability of lymphatic vessels,^4–7^ and/or impaired spontaneous contractions of the collecting lymphatic vessels that pump lymph centrally.^8,9^ The consequences of lymphatic dysfunction may also be more subtle. Recent studies suggest that altered lymphatic function contributes to subclinical edema that can compromise organ function under certain conditions, such as heart failure with preserved ejection fraction^10^ and diseases associated with the accumulation of tissue sodium.^11,12^

Subtle consequences of lymphatic dysfunction are also beginning to be appreciated in the context of metabolic diseases, including aging, obesity, type 2 diabetes, and metabolic syndrome. Lymphatic contractile dysfunction has been demonstrated in animal models of aging,^13,14^ obesity,^6^,^15–17^ TNFα overexpression,^18,19^ iNOS activation,^20,21^ metabolic syndrome,^21,22^ and other conditions associated with chronic inflammation.^23–25^ Lymphatic valve dysfunction, which impairs unidirectional lymph transport, is associated with animal models of obesity, with^6^ or without^26^ ApoE deficiency, and in models of TNFα hyperactivity.^18,27^ Lymphatic collecting vessel hyperpermeability, which counteracts the central return of lymph, has been demonstrated in animal models of obesity^5,6,26,28^ and in a model of type 2 diabetes.^29^ However, the mechanisms by which lymphatic dysfunction contributes to or results from the primary disease are not clear.

A common feature of these disease models is chronic metabolic stress. The primary source of intracellular ATP is the mitochondrial electron transport chain (ETC) and one mechanism of coupling the electrical activity of cells to their metabolic state is through the activity of ATP-sensitive K^+^ (K_ATP_) channels. K_ATP_ channels are voltage-independent, K^+^-selective channels that are activated by intracellular ADP and inhibited by intracellular ATP.^30,31^ By sensing the ATP/ADP ratio, they act as “metabolic sensors of the cell.”^32^ Mammalian K_ATP_ channels are hetero-octameric complexes comprised of four inward-rectifying K^+^ channel (Kir6) pore-forming subunits and four regulatory sulphonylurea receptor (SUR) subunits. Two pairs of genes (*ABCC8, KCNJ11*, and *ABCC9, KCNJ8*) on chromosome 11, each encode one pair of subunits—SUR1, Kir6.2, and SUR2, Kir6.1, respectively.^30^ The expression of different combinations of Kir6 and SUR subunits results in distinct K_ATP_ channel properties and functional roles in different cell types.^30,32^ In the vasculature, K_ATP_ channels normally have low activity in arterial smooth muscle cells but become activated under ischemic conditions,^30^ leading to arterial smooth muscle cell hyperpolarization and dilatation, which in turn increase blood flow and oxygen supply to the ischemic tissue.^32^ The composition of K_ATP_ channels expressed in lymphatic muscle is similar to that in arterial smooth muscle (Kir6.1 and SUR2B). Although K_ATP_-dependent vasodilation plays a protective role in arteries,^30,33,34^ similar depression of lymphatic muscle excitability by K_ATP_ channel activation could promote edema formation and sustain tissue inflammation as a consequence of impaired active lymph transport.^8,35^

In the present study, we hypothesized that acute mitochondrial inhibition would suppress ATP production and result in K_ATP_ channel activation, reducing lymphatic muscle excitability and impairing the active lymph pump. Furthermore, given that metabolic stress can increase ROS production in mitochondria,^36^ we also sought to evaluate the possible contribution of ROS. Our findings suggest that metabolic stress contributes to the contractile component of lymphatic dysfunction observed in metabolic diseases through ROS-dependent and -independent activation of K_ATP_ channels in lymphatic muscle.

## Methods

### Ethical Approval

All protocols and procedures were reviewed and approved by the Institutional Animal Care and Use Committee of the University of Missouri (protocol #9797) and performed in accordance with the National Institutes of Health’s Guide for the Care and Use of Laboratory Animals (8th edition, 2011).

### Mice

Mice were housed and bred under pathogen-free conditions in a controlled environment (22 ± 2°C, 12/12-hr light/dark cycle) of the animal facility of the University of Missouri School of Medicine. C57BL/6 wild-type (WT) mice were purchased at 5 weeks of age from Jackson Laboratory (C57Bl/6 J, strain #000 664, Bar Harbor, ME, USA). Kir6.1^−/−^ mice were a gift from Susumu Seino (Kobe University). Dominant negative Kir6.1 (*eGFP-Kir6.1[AAA]*) mice were described previously.^37^ Mice carrying eGFP-Kir6.1[AAA] were crossed to *Myh11-CreER^T2^* mice (JAX No. 01 979) to generate *Myh11-CreER^T2^; Kir6.1-AAA* mice with an inducible dominant negative Kir6.1 transgene expressed specifically in smooth muscle. The offspring were injected with tamoxifen (10 mg mL^−1^ in safflower oil) for consecutive 5 days and allowed to recover for 14 days; we confirmed the absence of GFP signal in the smooth muscle cell layer before testing. For ROS imaging, *Myh11-CreER^T2^; tdTomato* mice were used. Genotypes were confirmed by PCR with Taq DNA Polymerase Premix (Intact Genomics Catalog #3249). Mice were studied at 6-8 weeks of age from either sex, depending upon availability; in the case of *Myh11-CreER^T2^* mice, only males were used, as the transgene is located on the Y-chromosome; for other protocols mice of both sexes were used. For experiments, mice were anesthetized by i.p. injection using a mixture of ketamine/xylazine (100/10 mg kg^−1^ body weight) and euthanized by intracardiac injection of KCl.

### Vessel Isolation, Pressure Myography, and Data Acquisition

We have previously documented the reliability and reproducibility of contraction parameter tests for mouse popliteal lymphatic vessels studied ex vivo.^4,38,39^ For the isolation of mouse popliteal lymphatic vessels, a proximal-to-distal incision was made in the skin of the dorso-lateral thigh to expose the superficial saphenous vein. Afferent popliteal lymphatic vessels on either side of this vein were removed and transferred to a Sylgard dissection dish with Krebs buffer containing albumin. After pinning and cleaning, a vessel was cannulated using two glass micropipettes (40-50 µm, outer diameter), pressurized to 3 cmH_2_O and further cleaned of any remaining tissue in order to track diameter accurately. The vessel was shortened to a length that contained only one valve. Polyethylene tubing (PE-190) attached to the back of each micropipette holder was connected to a 2-channel microfluidic device (Elveflow OB1 MK3, Paris) for computer control of pressure on the stage of an inverted microscope. Input and output pressures were transiently set to 10 cmH_2_O immediately after set up and the vessel was stretched axially to approximate the in vivo length, which minimized longitudinal bowing and associated diameter-tracking artifacts during subsequent protocols.^40^ With input and output pressure held at 3 cmH_2_O, spontaneous contractions typically began within 15-30 min of warm-up and each vessel was allowed to stabilize at 37°C for 30-60 min before beginning an experimental protocol. A suffusion line connected to a peristaltic pump exchanged the chamber contents with Krebs buffer at a rate of 0.5 mL/min. A custom-written LabVIEW (National Instruments; Austin, TX, USA) algorithm^41^ measured the inner diameter of the vessel from video images obtained at 30 fps using a Basler A641fm firewire camera.

### Assessment of Lymphatic Contractile Function

Spontaneous contractions were recorded with equal input and output pressures (3 cmH_2_O) to prevent a pressure gradient for forward flow through the vessel during the experiment. The effects of mitochondrial ETC inhibitors or oxidative phosphorylation inhibitors on WT, *Kir6.1^−/−^* and *Myh11-CreER^T2^; Kir6.1[AAA]* vessels were determined by adding the compounds to the perfusate. At the end of the perfusion period (20-60 min), a small, predetermined volume of GLIB (1 µm) was added to the 3 mL bath, followed by thorough mixing while the bath was stopped. At the end of every experiment, all vessels were perfused with Ca^2+^-free Krebs buffer containing 3 m m EGTA for 30 min, and passive diameters were recorded at 3 cmH_2_O pressure (D_MAX_). After an experiment, custom-written analysis programs were used to detect peak end-diastolic diameter (EDD), end-systolic diameter (ESD), contraction amplitude (AMP), and contraction frequency (FREQ) on a contraction-by-contraction basis at rest before addition of the drug (FREQ_REST_) and following administration of drug.^42^ When FREQ was zero, no value of AMP was recorded. Fractional Pump Flow (FPF) is the best estimate of net flow and is a calculated variable due to the lack of flow meters with sensitivities in nL/min range. These data were used to calculate several commonly reported parameters that characterize lymphatic vessel contractile function. Each parameter was averaged over a 5 min period and used to calculate the following indices of lymphatic contractile function:


(1) \begin{eqnarray*}
{\mathrm{Contraction\,\,\textit{Amplitude}\,\,(AMP) = \,\,EDD - ESD}}
\end{eqnarray*}

(2) \begin{eqnarray*}
{\mathrm{Normalized\,\,\textit{Contraction}\,\,\textit{Amplitude} = \,\,}}\left( {\frac{{{\mathrm{EDD - ESD}}}}{{{{\mathrm{D}}_{{\mathrm{MAX}}}}}}} \right){\mathrm{ \times 100}}
\end{eqnarray*}

(3) \begin{eqnarray*}
{\mathrm{Ejection\,\,\textit{Fraction}\,\,(EF) = \,\,}}\left[ {\frac{{{\mathrm{ED}}{{\mathrm{D}}^{\mathrm{2}}}{\mathrm{ - ES}}{{\mathrm{D}}^{\mathrm{2}}}}}{{{\mathrm{ED}}{{\mathrm{D}}^{\mathrm{2}}}}}} \right]
\end{eqnarray*}

(4) \begin{eqnarray*}
{\mathrm{Fractional\,\,\textit{Pump}\,\,\textit{Flow}\,\,(FPF) = \,\,EF}} \cdot {\mathrm{FREQ}}
\end{eqnarray*}

(5) \begin{eqnarray*}
{\mathrm{Normalized\,\,\textit{Frequency} = \,\,}}\left( {\frac{{{\mathrm{FREQ}}}}{{{\mathrm{FRE}}{{\mathrm{Q}}_{{\mathrm{avg}}}}}}} \right){\mathrm{ \times 100}}
,
\end{eqnarray*}

where FREQ_avg_ represents the average frequency (in contractions per minute; cpm) during the baseline period before the addition of a drug to the bath and D_MAX_ represents the maximum passive diameter (obtained after incubation with calcium-free Krebs solution) at a given level of intraluminal pressure.

### V_m_ Measurement

Popliteal lymphatic vessels from WT mice were isolated and pressurized as described above. Wortmannin (1 µm; Tocris Bioscience, Bristol, UK) was applied to the perfusion bath for 30 min to inhibit myosin light chain kinase until contractions were sufficiently blunted to maintain impalements into lymphatic smooth muscle using intracellular microelectrodes (200-320 MΩ) filled with 1 M KCl solution. The preservation of small contractions (≤5 µm amplitude) allowed us to monitor the viability of the preparation. Membrane potential was sampled at 1-5 KHz using an AxoClamp2A amplifier, digitized through an A-D interface (USB-6216, National Instruments) and recorded using a custom LabVIEW program. Once impaled, V_m_ was allowed to stabilize before action potentials and multiple contraction cycles were recorded for analysis. Once recordings were completed, the electrode was retracted and the recording was corrected for any offset potential.

### Analysis of ROS Production in Lymphatic Muscle

Popliteal lymphatic vessels from tamoxifen-treated *Myh11-CreER^T2^; tdTomato* or WT mice were dissected and cannulated. To evaluate ROS production, the pressurized vessels were loaded with dihydrorhodamine 123 (DHR; Fisher Scientific), a membrane permeable dye that coverts to cationic rhodamine 123 upon oxidation and then localizes to mitochondria.^43^ DHR was dissolved in DMSO and diluted to 10 µm in Krebs buffer, perfused and preincubated for 10 min in a bath and remained in the superfusion solution throughout the experiment. All vessels were incubated at 37°C in a light-protected environment. Nifedipine (1 µm) was applied to the bath to completely inhibit any spontaneous contractions that otherwise would have prevented maintaining focus on the smooth muscle layer. Fluorescence images were acquired for 100 ms at 1 min intervals for 15 min with 10× (Olympus UPlanApo N.A. = 0.40) or 20× (Olympus UPlanSApo N.A. = 0.75) objectives coupled to an EMCCD camera (Photometrics Cascade II) on an Olympus IX81 inverted microscope. Illumination was provided by an Andor/Yokogawa CSU-X Confocal Spinning Disk system with excitation at 472/30 nm and emission at 525/35 nm. Fluorescence intensity was quantified with ImageJ (National Institutes of Health) in a region of interest located in the middle of a vessel following subtraction of background fluorescence. As a positive control for generating ROS, the mitochondria complex III inhibitor, antimycin A (1 µm) was added to the superfusion solution.^44^ To verify the sensitivity of DHR to endogenous ROS production, experiments were repeated following 10 min of preincubation with tiron (1 m m) in combination with polyethylene glycol (PEG)-catalase 250 U mL^−1^. The respective reagents were present throughout the rest of the experiment. Values for DHR fluorescence are expressed in arbitrary units for the change from baseline within a ROI (△ = fluorescence at x min − fluorescence at 0 min, where x represents 1 min intervals during chemical exposure).^45^

### Solutions and Chemicals

Krebs buffer contained (in m m) 146.9 NaCl, 4.7 KCl, 2 CaCl_2_·2H_2_O, 1.2 MgSO_4_, 1.2 NaH_2_PO_4_·H_2_O, 3 NaHCO_3_, 1.5 NaHEPES, and 5 D-glucose (pH = 7.4). Krebs-BSA buffer was prepared with the addition of 0.5% bovine serum albumin. During cannulation, Krebs-BSA buffer was present both luminally and abluminally, but during the experiment the bath solution was replaced with Krebs solution without albumin. Ca^2+^ free Krebs buffer was used at the end of experiment to obtain the maximum passive diameter. All chemicals and drugs (rotenone, antimycin A, CCCP, tiron, PEG-catalase) were purchased from Sigma-Aldrich (St. Louis, MO, USA), with exception of BSA (United States Biochemicals; Cleveland, OH, USA), MgSO_4_, Na-HEPES (ThermoFisher Scientific; Pittsburgh, PA, USA). PEG-catalase was dissolved in distilled water. Antimycin A, CCCP, rotenone and GLIB were dissolved in DMSO and the total amount of DMSO was set below 0.4%, which was determined in separate protocols to be the vasoactive threshold concentration.

### Statistical Procedures

The number *n* refers to the total number of vessels included per group as stated in the figure legends; *N* is number of animals. Values are means ± SD. Statistical analysis was undertaken only for studies where each group size ≥5. Randomization was not a feature of study design. Because baseline values of spontaneous contraction frequency are highly variable among lymphatic vessels,^46^ we expressed the data as normalized values of FREQ, AMP, or FPF, with each value normalized to the average of the respective control values. Differences between FREQ, AMP, or FPF in the presence or absence of treatment were assessed using ANOVA or paired Student’s *t*-tests as stated in the figure legends. All statistical analyses were performed using Prism9 (GraphPad Software Inc., CA, USA), with significance for all tests set at *P* < 0.05.

## Results

### Mitochondrial ETC Disruption Impairs Lymphatic Pacemaking

**Figure 1.**
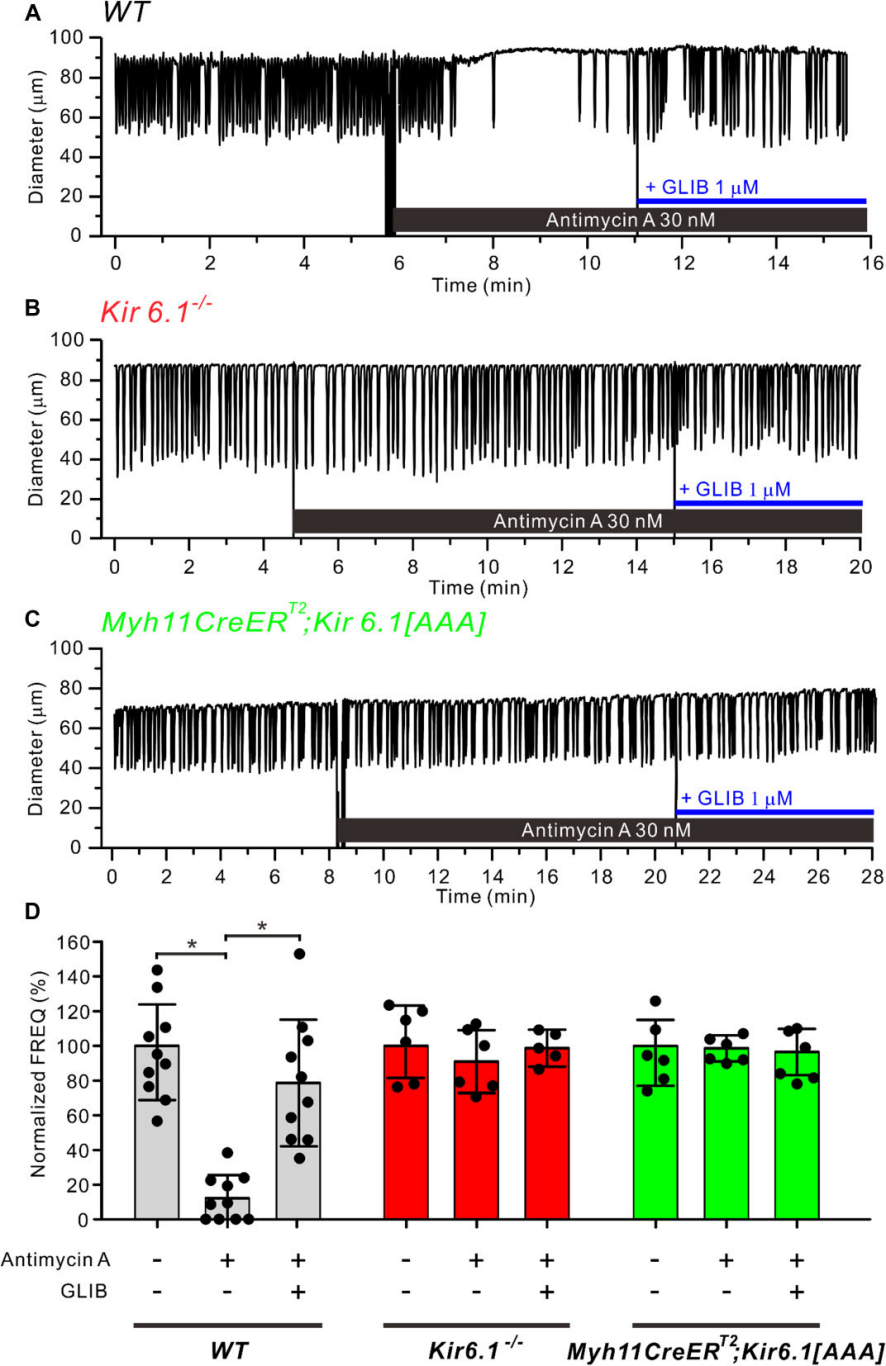
Antimycin A reduces lymphatic muscle contraction frequency and the inhibition is reversed by GLIB. Responses of mouse popliteal lymphatics, pressurized to 3 cmH_2_O, exposed to the mitochondrial ETC complex III inhibitor, antimycin A (30 n m). Example recordings of vessels from (**A**) wild-type (WT), (**B**) *Kir6.1^−/−^* and (**C**) *Myh11-CreER^T2^; Kir6.1[AAA]* mice. During the last 2 min of the recording period, the frequency of only the WT vessel was reduced by antimycin A and that reduction was largely rescued by GLIB (1 µm). (**D**) Summary of changes in normalized frequency for the different treatments and genotypes. Gray bars represent WT vessels (*N* = 7; *n* = 10), red bars are *Kir6.1^−/−^* vessels (*N* = 6; *n* = 5) and green bars are *Myh11-CreER^T2^; Kir6.1[AAA]* vessels (*N* = 4; *n* = 6). All data are means ± SD. *P < 0.05 between WT vessels before and after treatment of antimycin A and GLIB using a one-way ANOVA with Tukey’s post-hoc tests.

When pressurized and heated to physiological intraluminal pressure and temperature, WT popliteal lymphatics developed spontaneous twitch contractions. At 37°C and 3 cmH_2_O intraluminal pressure, the typical lymphatic contraction pattern shown at the start of the recording in Figure 1A was stable for hours, although in some vessels occasional patterns of burst contractions developed over time. In this example, the contraction amplitude was ∼40 µm (41% of maximal passive diameter) and frequency was 11 contractions per minute (cpm) until ∼1 min after the application of the ETC complex III inhibitor antimycin A (30 n m). This concentration was slightly higher than the IC_50_ (12.2 n m) used to inhibit mitochondrial oxygen consumption.^47^ In the presence of antimycin A, contractions nearly ceased during the last 5 min of antimycin A treatment [control FREQ = 11 cpm vs 1 cpm (12.2% of control)]. Contraction amplitude was largely unaffected even after 20 min exposure to antimycin A. The subsequent addition of GLIB (1 µm), at a concentration that we found previously could reverse impaired contractions in vessels from mice expressing overactive K_ATP_ channels,^8,35^ partially restored contraction frequency. Both 3 and 10 µm GLIB produced more complete recovery of frequency (not shown), but here we used 1 µm, a concentration that minimizes off-target effects associated with higher concentrations of this inhibitor.^48^

Under baseline conditions, popliteal lymphatics from *Kir6.1^−/−^* mice developed similar patterns of spontaneous contractions as WT mice (Figure 1B). The lower basal contraction frequency in this particular vessel reflects natural vessel-to-vessel variation and was not a consistent feature of *Kir6.1^−/−^* vessels,^35,48^ as K_ATP_ channels make little contribution to the basal excitability of lymphatic muscle cells (LMCs) of mice under normal (normoxic) conditions. In contrast to the WT vessel, the *Kir6.1^−/-^*vessel was almost completely resistant to the effects of antimycin A [control FREQ = 6.8 cpm vs 5.7 cpm during the last 5 min of antimycin A] and subsequent addition of GLIB (1 µM) produced little further effect (FREQ = 6.2 cpm). Although we previously demonstrated that functional K_ATP_ channels are expressed in smooth muscle but not endothelium of mouse popliteal vessels, here we expressed a dominant negative construct of Kir6.1[AAA]^37^ specifically in the muscle layer to confirm that LMC K_ATP_ channels were responsible for protection from the effects of antimycin A. The *Myh11-CreER^T2^; Kir6.1[AAA]* contraction pattern was similar to the WT vessel prior to administration of antimycin A, (Figure 1C). As with the *Kir6.1^−/−^* vessel, the *Myh11-CreER^T2^; Kir6.1[AAA]* vessel was resistant to the effects of antimycin A (control FREQ = 10.3 cpm vs 10.3 cpm after antimycin A) and the subsequent application of GLIB (1 µm) had little additional effect (FREQ = 9.9 cpm). These observations suggest that treatment with antimycin A results in the activation of K_ATP_ channels in the muscle layer of mouse popliteal lymphatic vessels to impair pump function.

Summary data for the effects of antimycin A ± GLIB on lymphatic vessels from the three genotypes are presented in Figure 1D. The frequency of WT vessels was significantly reduced by antimycin A and that depression of frequency was largely reversed by GLIB (1 µm), as predicted if the mechanism involved activation of K_ATP_ channels. Antimycin A produced a small but nonsignificant increase in normalized AMP in WT vessels that also was restored to control levels by GLIB (Suppl. Figure 1A). The reduction in FREQ (Figure 1D) was more than sufficient to offset the increase in normalized AMP and produce a significant reduction in calculated fractional pump flow (FPF) (Suppl. Figure 1B), which is an estimate of active lymph transport. The antimycin A-induced reduction in normalized FPF of WT vessels was significantly rescued by GLIB (Suppl. Figure 1B). Antimycin A had no significant effects on the normalized amplitude or FPF of *Kir6.1^−/−^* vessels or *Myh11CreER^T2^; Kir6.1[AAA]* vessels (Suppl. Figure 1A, B).

**Figure 2.**
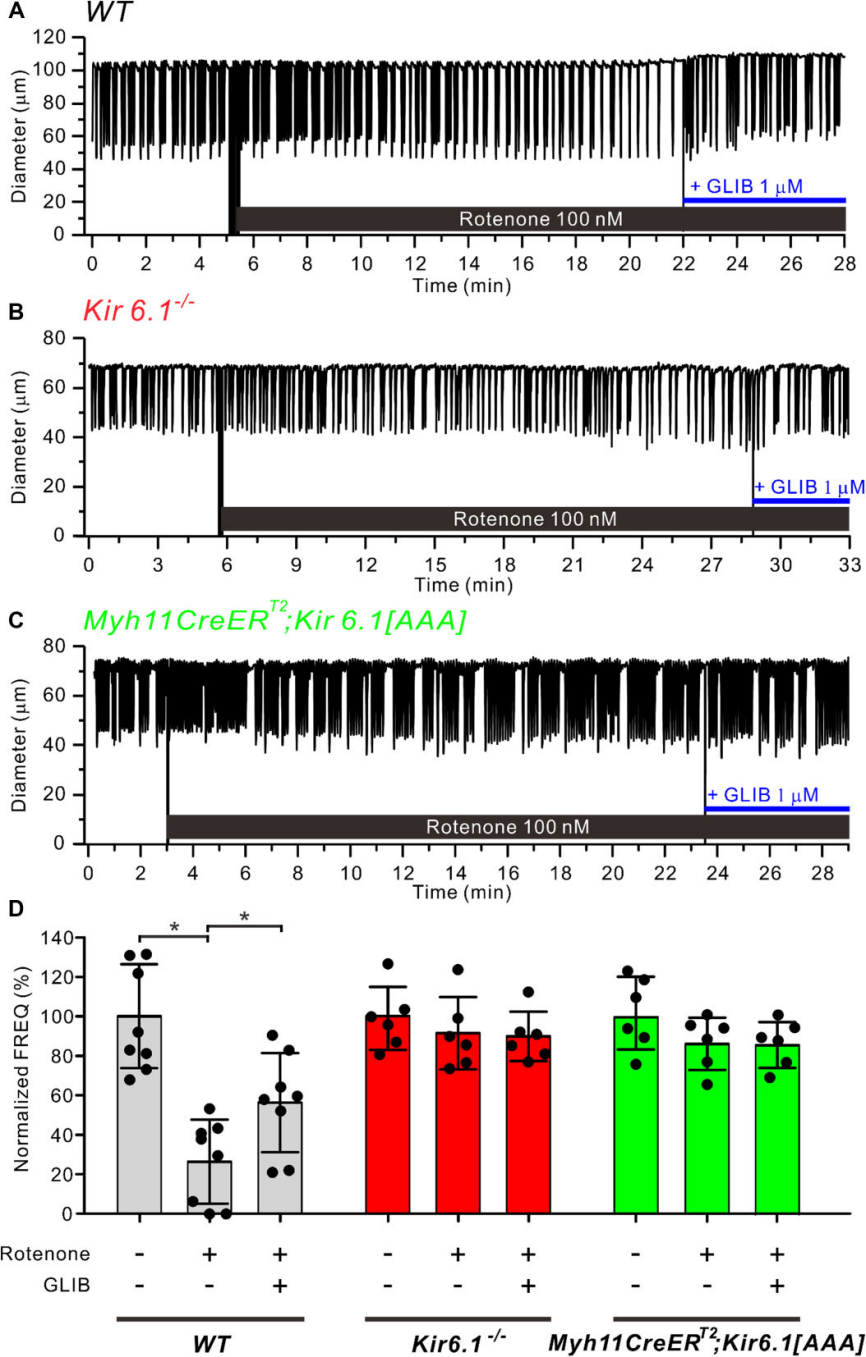
Rotenone reduces lymphatic muscle contraction frequency and the inhibition is reversed by GLIB. Representative examples of the effects of the mitochondrial ETC complex I inhibitor, rotenone (100 n m) on spontaneous contractions of mouse popliteal lymphatic vessels from (**A**) WT, (**B)**  *Kir6.1^−/^*^−^, and (**C**) *Myh11-CreER^T2^; Kir6.1[AAA]* mice. The frequency of only the WT vessel was reduced by rotenone and this effect was largely rescued by GLIB (1 µm). (**D**) The summary graphs showing normalized frequency for the various treatments and genotypes. Gray bars represent WT vessels (*N* = 6; *n* = 8), red bars are *Kir6.1^−/−^* vessels (*N* = 3; *n* = 6) and green bars are *Myh11-CreER^T2^; Kir6.1[AAA]* vessels (*N* = 3; *n* = 6). All data are means ± SD. *P < 0.05 between WT vessels before and after treatment of rotenone and GLIB using a one-way ANOVA with Tukey’s post-hoc tests.

Next, we explored the actions of a different metabolic stressor, rotenone, which inhibits ETC complex I.^49^ Rotenone (100 n m) caused a progressive decline in the frequency of spontaneous lymphatic contractions, from a control FREQ = 9.9 cpm to 2.2 cpm (22.2% of control) during the last 5 min of rotenone treatment, which was reversed by GLIB (1 µm) (Figure 2A). Note that this vessel had a bursting contraction pattern, whereby 3-8 contractions occurred in rapid succession followed by a short pause prior to the next contraction burst. In contrast, a popliteal lymphatic from a *Kir6.1^−/−^* mouse was largely resistant to the effects of rotenone, with control FREQ = 8.1 cpm vs 7.6 cpm during the last 5 min of rotenone treatment; subsequent GLIB (1 µm) application had little additional effect (Figure 2B). A popliteal lymphatic from a *Myh11-CreER^T2^; Kir6.1[AAA]* mouse (also with a bursting contraction pattern) was also resistant to rotenone (control FREQ = 14.1 cpm vs 13.1 cpm during the last 5 min of rotenone) (Figure 2C). Summary data for the effects of rotenone (100 n m) on the 3 genotypes are plotted in Figure 2D. The only significant effects of rotenone were on WT vessels, in which it lowered frequency to ∼25% of control, with partial rescue by GLIB (1 µm). Rotenone had no significant effects on normalized AMP for any of the three genotypes (Suppl. Figure 1C). Rotenone significantly reduced the normalized FPF of WT vessels, but not the normalized FPF of Kir6.1^−/−^ vessels or *Myh11-CreER^T2^; Kir6.1[AAA]* vessels (Suppl. Figure 1D).

**Figure 3.**
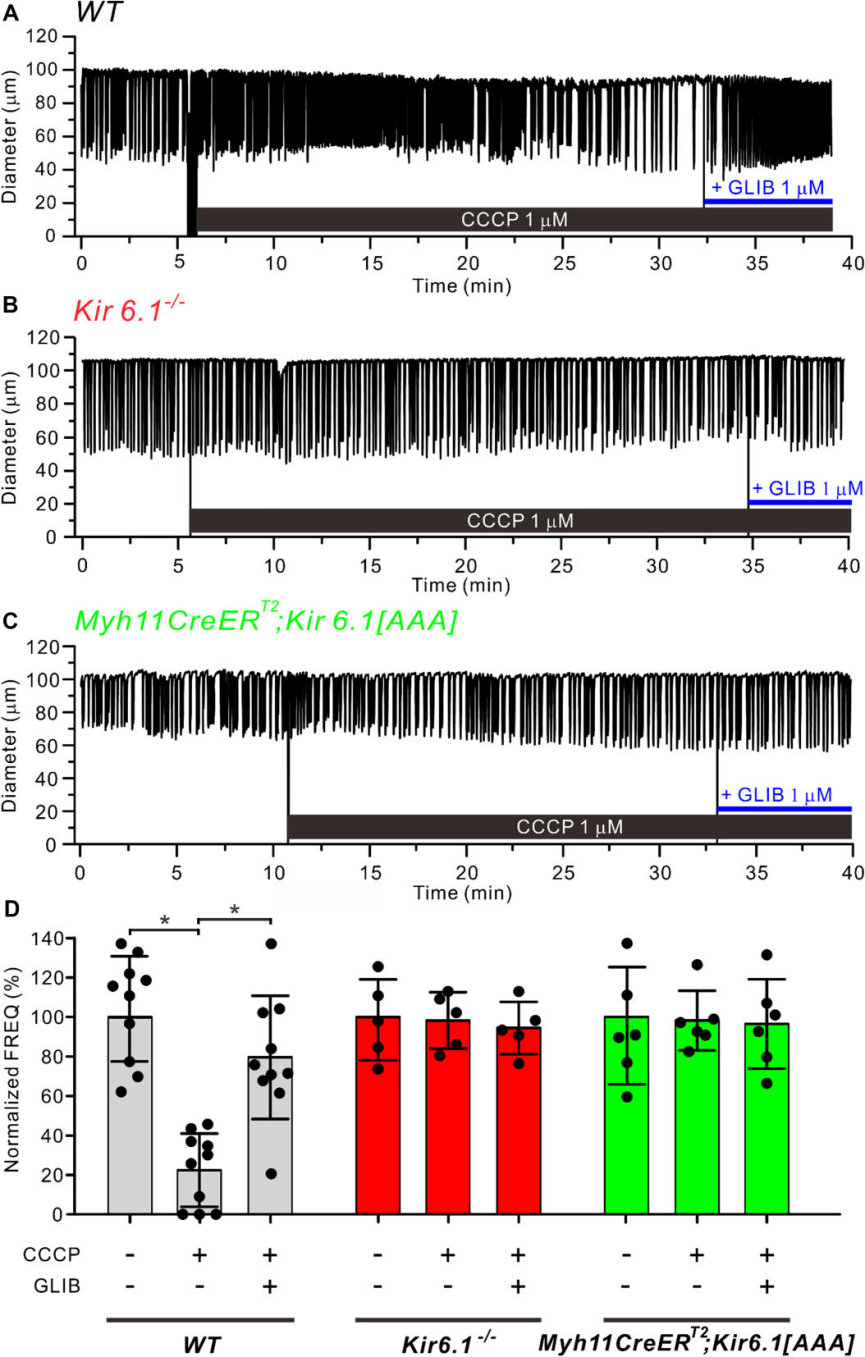
CCCP reduces lymphatic muscle contraction frequency and the inhibition is reversed by GLIB. Representative examples of the effects of the oxidative phosphorylation inhibitor, CCCP (1 µm), on mouse popliteal lymphatics from (**A**) WT, (**B**) *Kir6.1^−/−^* and (**C**) *Myh11-CreER^T2^; Kir6.1[AAA]* mice. The frequency of only the WT vessel was reduced by CCCP; it was largely rescued by GLIB (1 µm). (**D**) Summary graphs showing normalized frequency for the various treatments and genotypes. Gray bars represent WT vessels (*N* = 6; *n* = 10), red bars are *Kir6.1^−/−^* vessels (*N* = 3; *n* = 6) and green bars are *Myh11-CreER^T2^; Kir6.1[AAA]* vessels (*N* = 3; *n* = 6). All data are means ± SD. *P < 0.05 between WT vessels before and after treatment of CCCP and GLIB one-way ANOVA with Tukey’s post-hoc tests.

Next, we tested the effects of the protonophore CCCP (1 µm), which reduces ATP production by uncoupling mitochondrial oxidative phosphorylation.^50^ The application of CCCP (1 µm) to a WT popliteal lymphatic lowered frequency from 9 cpm in the control period to 2 cpm (22.2% of control) during the last 5 min of CCCP (Figure 3A), and the effect was reversed by GLIB (1 µm). In contrast, popliteal vessels from a *Kir6.1^−/−^* mouse (Figure 3B) and an *Myh11-CreER_T2_; Kir6.1[AAA]* mouse (Figure 3C) were resistant to the effects of CCCP. The data for CCCP on normalized frequency are summarized for the three genotypes in Figure 3D. The only significant effects of CCCP were on WT vessels, which on average lowered frequency to 22% of control and showed partial rescue by GLIB (1 µm). There were no significant changes in normalized amplitude in response to CCCP for any of the three genotypes, suggesting there was still sufficient ATP for normal actomyosin crossbridge cycling (Suppl. Figure 1E). As a consequence of the reduction in frequency, CCCP caused a significant reduction in the normalized FPF of WT vessels (but not *Kir6.1^−/−^* vessels or *Myh11-CreER^T2^; Kir6.1[AAA]* vessels) (Suppl. Figure 1F).

### Complex III Inhibition Suppresses Lymphatic Action Potentials

**Figure 4.**
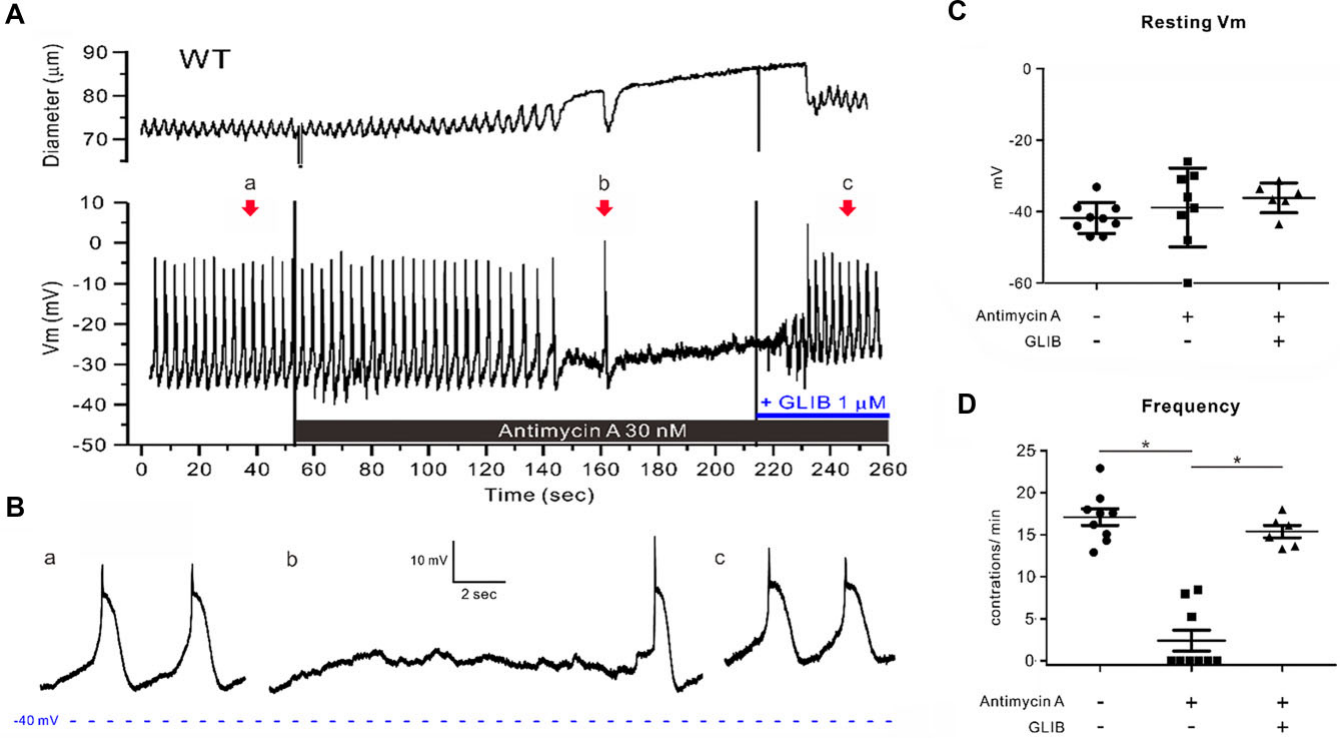
Antimycin A inhibits action potentials in LMCs and GLIB counteracts its effects. Raw traces showing that antimycin A (30 n m) inhibits the frequency of spontaneous lymphatic contractions (**A**) and action potentials (APs). Membrane potential (V_m_) was recorded using an intracellular electrode in a LMC of a WT mouse popliteal lymphatic vessel pressurized at 3 cmH_2_O. The recording was made in the presence of 1 µm wortmannin to minimize vessel wall movement. (**B**) Magnification of APs in panel A prior to drug (**a**), in the presence of antimycin A (**b**), and antimycin A and GLIB (1 µm), which restored AP generation (**c**). (**C**) Summary of resting V_m_ values in lymphatic muscle cells before and after treatment of antimycin A and/or GLIB (*N* = 8; *n* = 9). (**D**) Summary of changes in AP (and concomitant contraction) frequency before and after treatment of antimycin A and GLIB. All data are means ± SD. *P < 0.05 using a one-way ANOVA with Tukey’s post-hoc tests.

Given these findings, we then tested the effects of antimycin A on the membrane potential of popliteal lymphatics from WT mice. Previously, we showed that K_ATP_ channel activation leads to inhibition of the ionic pacemaker underlying spontaneous lymphatic contractions, but that overt hyperpolarization is not required to inhibit action potential generation.^35,51^ Such was the case for antimycin A. Approximately 100 sec after antimycin A application, spontaneous action potentials ceased, even though the resting V_m_ depolarized slightly (Figure 4A), contractions stopped and the vessel dilated. The inhibition of action potential firing despite slight depolarization suggests that the threshold for action potential firing was reset^52^ during this period (Figure 4B, insert b). The effect of this concentration of antimycin A was similar to that produced by 100-300 n m pinacidil in previous studies.^48,51^ Summary V_m_ data for nine LMCs from nine different vessels showed that spontaneous action potential generation was abolished by antimycin A in most cells without significant hyperpolarization (with one exception) (Figure 4C). GLIB (1 µm) application restored AP generation in all cells (Figure 4D). Although we did not perform Vm measurements during rotenone or CCCP application, there is a 1:1 correspondence of twitch contractions with APs,^35,53,54^ and the contraction recordings for rotenone and CCCP suggest that both drugs also inhibited AP firing.

### ROS Contribution to Metabolic Disruption of Lymphatic Pacemaking

**Figure 5.**
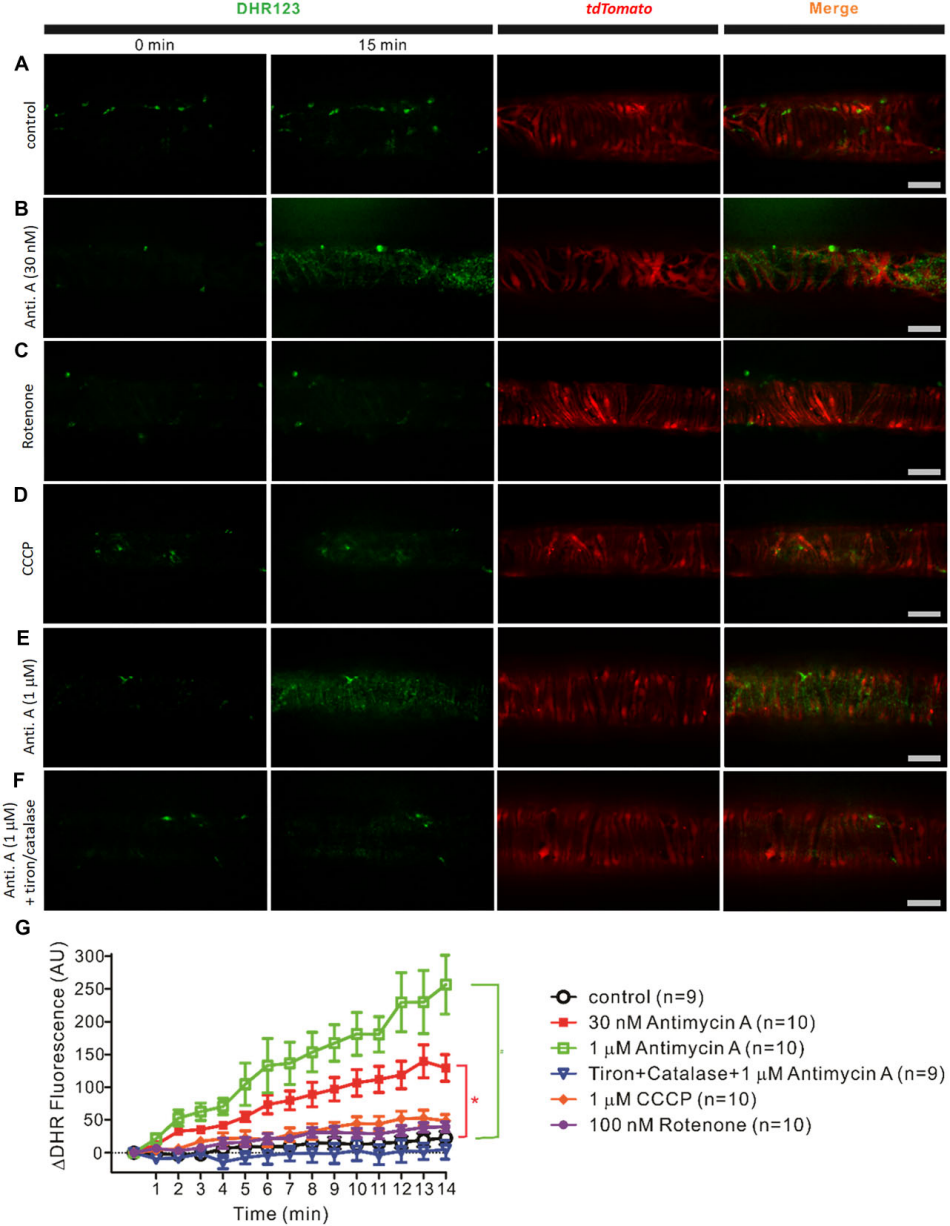
Antimycin A, but not rotenone or CCCP enhance ROS generation in WT lymphatic vessels. Nifedipine 1 µm was used to block lymphatic contractions of pressurized (3 cmH_2_O) popliteal lymphatic vessels from *Myh11-CreER^T2^; tdTomato* mice so that the microscope objective could be focused on the tdTomato signal in the LMC layer. Representative images from untreated lymphatic vessels (**A**; *N* = 9; *n* = 9) and lymphatic vessels treated with 30 n m antimycin A (**B**; *N* = 9; *n* = 10), 100 n m rotenone (**C**; *N* = 6; *n* = 10), 1 µm CCCP (**D**; *N* = 7; *n* = 10), 1 µm antimycin A (**E**; *N* = 9; *n* = 10), and 1 µm antimycin A + 1 m m tiron and 250 U/mL catalase (**F**; *N* = 9; *n* = 9) showing initial DHR fluorescence (0 min; First column), 15 min of DHR accumulation (Second column), the td-tomato signal (Third column) and the merged signal (Fourth column) from columns 2 and 3. Scale bars are 50 µm. (**G**) Summary data for changes for in DHR123 fluorescence over a 15 min measurement period. Values are means ± SD tested with a one-way ANOVA and Dunnett's post-hoc test. *P < 0.05 antimycin A (30 n m or 1 µm) vs control. ^#^*P* < 0.05 antimycin A (1 µm) + tiron + catalase vs antimycin A (1 µm).

Next, we measured production of ROS in the muscle layer using DHR. Popliteal lymphatics expressing the td-Tomato reporter in LMCs under the control of *Myh11CreER^T2^* enabled us to focus on the fluorescence signal in the LMC layer per se, while nifedipine (1 µm) was used to completely block any wall movement associated with spontaneous contractions. The DHR signal in the focal plane of the LMCs is shown in the first 2 columns of Figure 5 (at 0 and 15 min), with the td-Tomato signal in the third column and the merged signal in the fourth column. A control vessel is shown in row **A**, a vessel treated with antimycin A (30 n m) in row **B**, a vessel treated with rotenone (100 n m) in row **C**, a vessel treated with CCCP (1 µm) in row **D**, a vessel treated with antimycin A (1 µm) in row **E**, and a vessel treated with antimycin A (1 µm) after pretreatment with tiron (1 m m) and Catalase (250 U/mL) in row **F**. The time courses of the DHR signals under these various conditions are plotted in Figure 5G. Both 30 n m and 1 µm antimycin A produced significant increases in DHR signal, and the latter was prevented by tiron and catalase pretreatment. Neither rotenone nor CCCP treatment led to a significant increase in ROS production.

**Figure 6.**
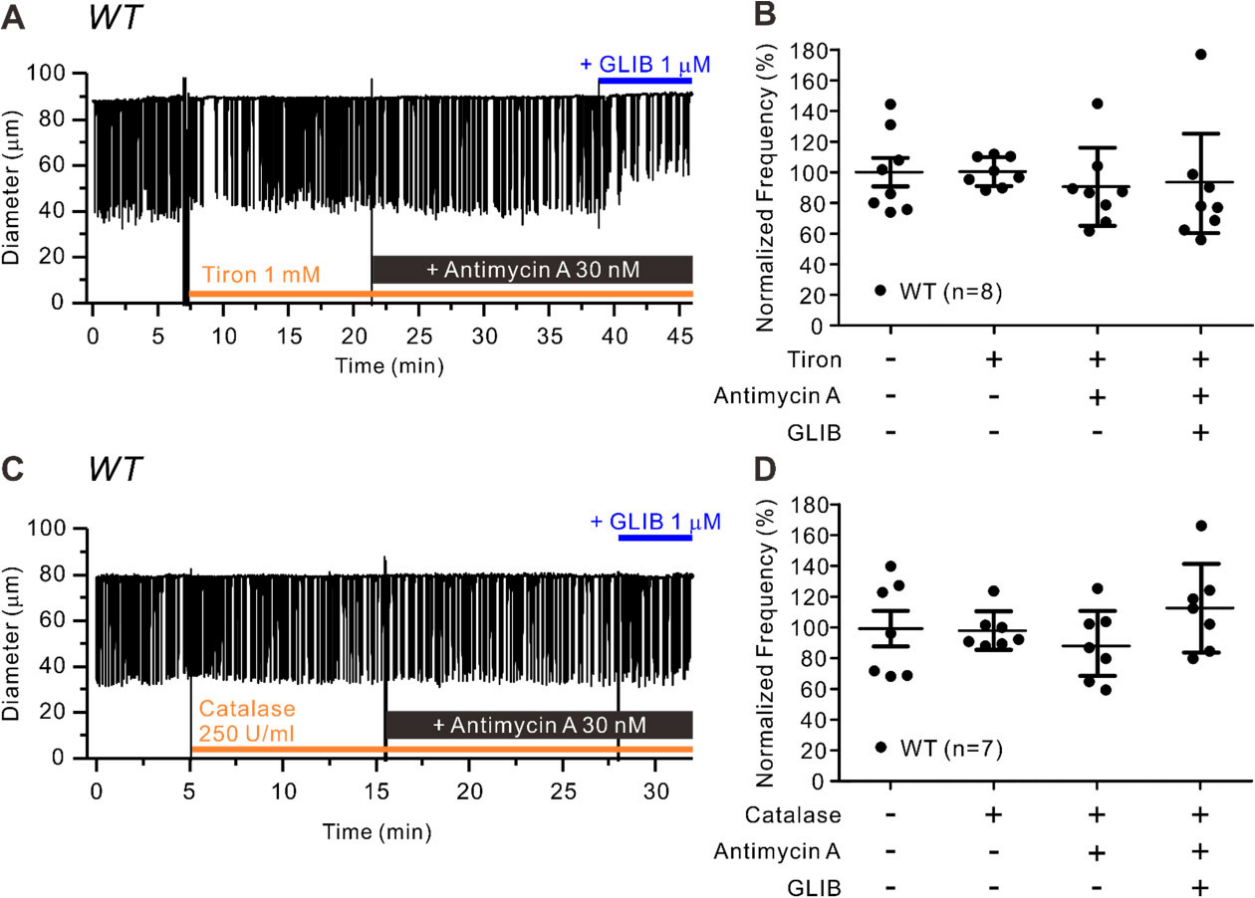
Scavenging ROS prevents the effects of antimycin A on lymphatic contraction. Raw traces of lymphatic contractions in WT vessels in response to antimycin A (30 n m) after pretreatment with (**A**) tiron (1 m m) or (**B**) catalase (250 U/mL) for 10 min and after subsequent addition of GLIB (1 µm). Summary of normalized frequency changes to antimycin A with and without **C**) tiron (*N* = 6; *n* = 8) and (**D**) catalase (*N* = 4; *n* = 7). All data are means ± SD. *P < 0.05 before and after treatment of rotenone and GLIB using a one-way ANOVA with Tukey’s post-hoc tests.

Having established that antimycin A induces ROS production in LMCs, we then asked whether pretreatment with ROS scavengers could prevent the effects of antimycin A on FREQ and FPF of WT lymphatic vessels. The example in Figure 6A shows a vessel treated with tiron (1 m m), at a concentration used to effectively scavenge O_2_^−^ in other studies.^55,56^ Tiron was applied to the bath for 10 minutes prior to the addition of antimycin A. In this case, a transient slowing of FREQ occurred almost immediately after the addition of tiron, but FREQ subsequently returned to normal (within 5 min). The application of antimycin A did not produce any substantial changes in contraction FREQ, nor did subsequent addition of GLIB (1 µm), which in this case reduced contraction amplitude by ∼40% (Figure 6A). The effect of GLIB on amplitude in this vessel was not a consistent one, as shown in the summary data in Suppl. Figure 2A. Tiron pretreatment effectively blocked the inhibitory effect of antimycin A on normalized FREQ (Figure 6B), but again without significant effects on either normalized AMP or FPF (Suppl. Figure 2A, B). A similar protocol was used to test the effects of the H_2_O_2_ scavenger catalase (250 U/mL),^55,56^ which also completely blocked the inhibitory effects of antimycin A on normalized FREQ (Figure 6C). Summary data for the effects of catalase pretreatment on effects of antimycin A confirm that there were no significant effects on either normalized FREQ (Figure 6D) or on AMP or FPF (Suppl. Figure 3A, B). These effects of tiron and catalase are consistent with the conclusion that antimycin A is producing ROS, which activates K_ATP_ channels to inhibit pacemaking frequency.

**Figure 7.**
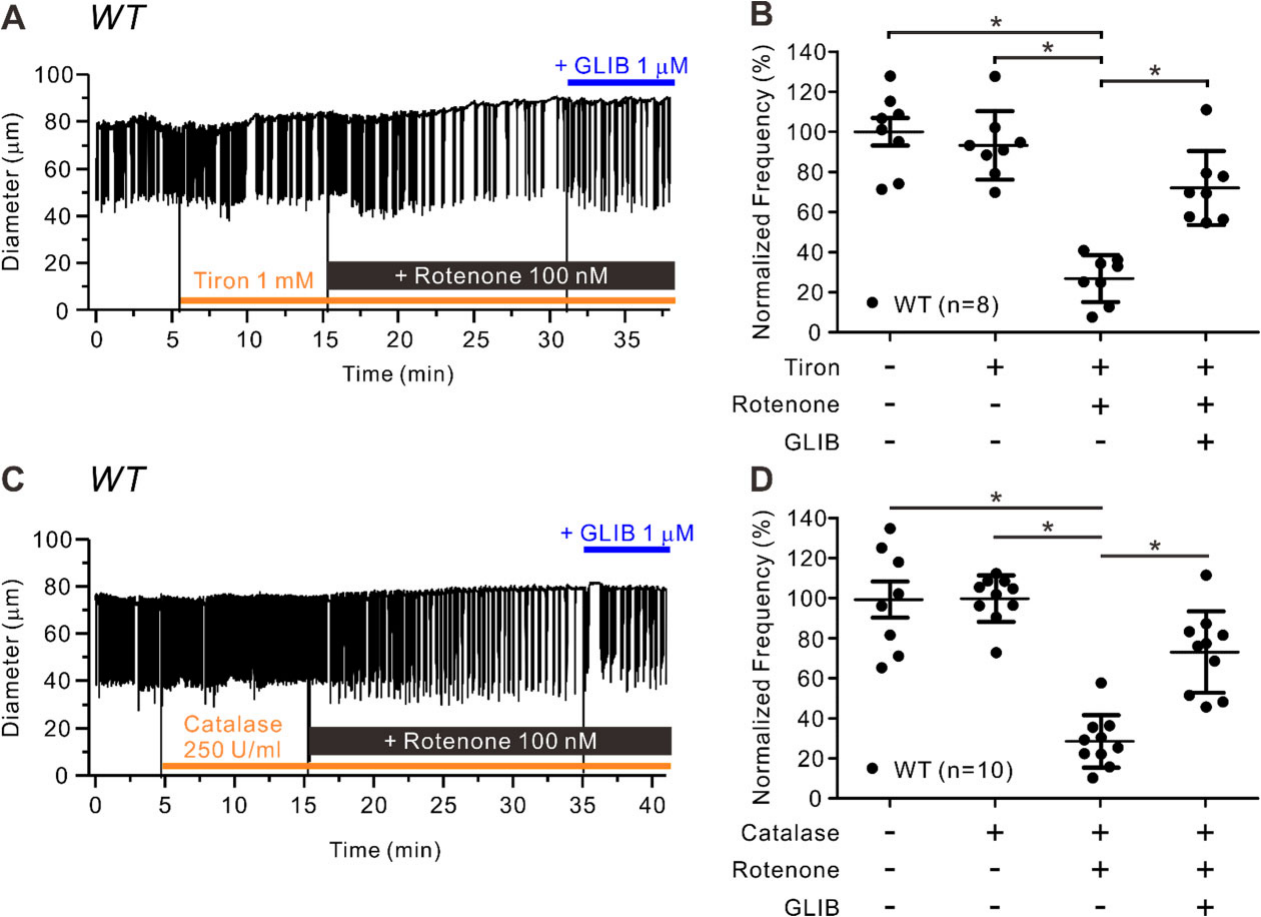
ROS scavengers do not prevent effects of rotenone on lymphatic contraction. Raw traces showing inhibition of lymphatic contraction frequency in WT vessels in response to rotenone (100 n m) after pretreatment with m (**A**) tiron (1 m m) or (**B**) catalase (250 U/mL) for 10 min and after subsequent addition of GLIB (1 µm). Summary of normalized frequency changes to rotenone with and without **C**) tiron (*N* = 5; *n* = 8) or (**D**) catalase pretreatment (*N* = 7; *n* = 10). All data are means ± SD. *P < 0.05 before and after treatment of rotenone and GLIB using a one-way ANOVA with Tukey’s post-hoc tests.

Similar protocols were conducted to test tiron and catalase pretreatment on the effects of rotenone. As shown in the example recording in Figure 7A, pretreatment with tiron failed to prevent the subsequent reduction in contraction FREQ in response to rotenone [control FREQ = 12.5 cpm vs 3.6 cpm (26.9% of control) during last 5 min of rotenone], as confirmed by the summary data in Figure 7B. Rotenone did not significantly inhibit normalized AMP (AMP actually increased) in the presence or absence of tiron (Suppl. Figure 2C) and tiron pretreatment failed to block rotenone-induced inhibition of normalized FPF (Suppl. Figure 2D). Likewise, pretreatment with catalase did not prevent the subsequent rotenone-induced reduction in contraction frequency [Figure 7C; control FREQ = 10.6 cpm vs 2.8 cpm (28.2% of control) during last 5 min of rotenone], as confirmed by the summary data in Figure 7D. Catalase pretreatment did not significantly alter the effect of rotenone on normalized contraction AMP (Suppl. Figure 3C) and failed to block the rotenone-induced inhibition of normalized FPF (Suppl. Figure 3D). These results confirm that rotenone does not inhibit lymphatic pumping through the production of ROS.

**Figure 8.**
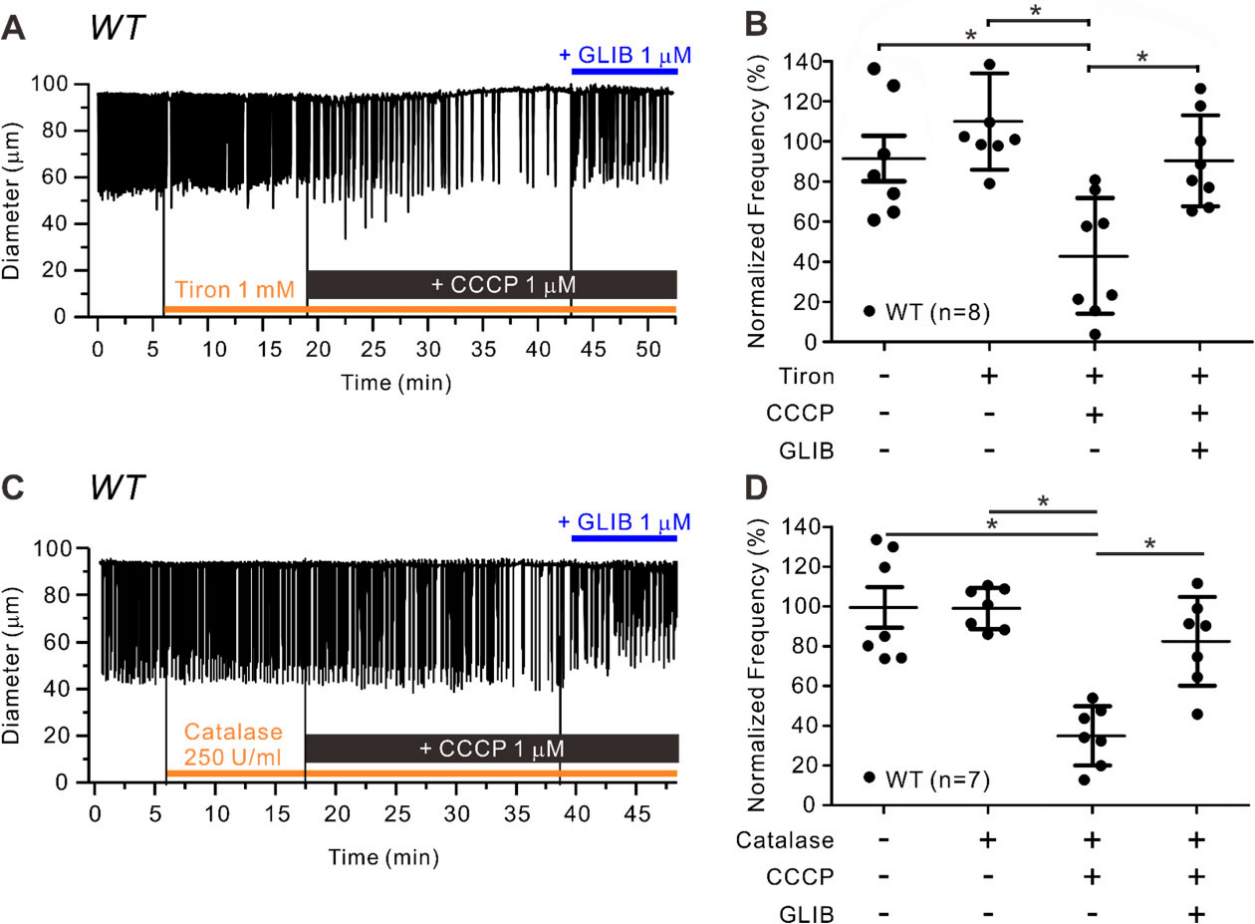
ROS scavengers do not prevent effects of CCCP inhibition on lymphatic contraction. Representative traces of lymphatic contractions in WT vessels in response to CCCP 1 µm pretreated with (**A**) tiron (1 m m) or (**B**) catalase (250 U/mL) for 10 min and after subsequent addition of GLIB (1 µm). Summary of normalized frequency changes to CCCP with and without **C**) tiron (*N* = 5; *n* = 8) or **D**) catalase (*N* = 4; *n* = 7) pretreatment. All data are means ± SD. *P < 0.05 before and after treatment of CCCP and GLIB using a one-way ANOVA with Tukey’s post-hoc tests.

Tiron pretreatment also failed to block the inhibitory effect of CCCP on contraction FREQ (Figure 8A; control FREQ = 10.5 cpm vs 4.6 cpm (42.4% of control) during the last 5 min of CCCP], but the inhibition was rescued by subsequent addition of GLIB (1 µm). The summary data in Figure 8B confirm that significant CCCP-induced inhibition of contraction FREQ persisted in the presence of tiron. Tiron did not alter the effect of CCCP on AMP and failed to prevent the CCCP-induced reduction in normalized FPF (Suppl. Figure 2E, F). Catalase likewise failed to block the inhibitory effect of CCCP on normalized FREQ [Figure 8C; control FREQ = 10.2 cpm vs 4.3 cpm (39.3% of control) during last 2 min of CCCP], but the inhibition was rescued by subsequent addition of GLIB (1 µm). The summary data in Figure 8D indicate that the reduction in normalized FREQ was significant. Catalase pretreatment did not alter the effect of CCCP on contraction AMP (Suppl. Figure 3E) but failed to prevent the CCCP-induced reduction in normalized FPF (Suppl. Figure 3F). These results suggest that CCCP does not inhibit lymphatic contraction through the production of ROS.

## Discussion

Our study is the first to evaluate the effects of metabolic inhibitors on K_ATP_ channels in lymphatic muscle. Three different inhibitors of mitochrondrial ATP production, two that inhibited the ETC and one that inhibited oxidative phosphorylation, each retarded the ionic pacemaker that drives spontaneous lymphatic contractions and active lymph pumping. The inhibitor of ETC complex III, antimycin A, acted through the production of ROS, as its effects on contraction frequency were prevented by pretreatment with either of two ROS scavengers, tiron and catalase. The ETC complex I inhibitor (rotenone) and the oxidative phosphorylation inhibitor CCCP did not increase ROS production and their effects were not blocked by ROS scavengers, consistent with the conclusion that K_ATP_ channel activation in these cases was mediated by a decrease in the intracellular ATP/ADP ratio.^57^ Three important findings from our study were that: (1) the effects of all three metabolic inhibitors on contraction frequency and pumping could be reversed by a relatively low and selective concentration of the K_ATP_ channel inhibitor GLIB; (2) ETC inhibition caused a selective reduction in contraction frequency without a concomitant inhibition of amplitude; and (3) mice lacking functional K_ATP_ channels in lymphatic muscle (*Kir6.1^−/−^* or *Myh11-CreER^T2^; Kir6.1[AAA]* mice) were completely resistant to the effects of the metabolic inhibitors. Further confirmation that the inhibitors worked through activation of K_ATP_ channels in lymphatic muscle came from direct measurements of V_m_ in LMCs in which antimycin A stopped the generation of spontaneous action potentials, and that inhibition was reversed by GLIB.

The effects of ETC inhibition tested in our study were intended to simulate the chronic metabolic stress associated with metabolic disease. Obesity and insulin resistance are linked to decreased efficiency of ATP synthesis and an increase in mitochondrial ROS production.^36^ This imbalance of energy production vs utilization is a hallmark of metabolic syndrome. While direct measures of ROS production in lymphatic vessels in animal models of metabolic disease in response to metabolic disorders are lacking, metabolic dysfunction induced by high fat or Western-style diets (ie, high fat and processed carbohydrate) are known to increase ROS production in skeletal muscle and cerebral artery smooth muscle.^45,58^ A key finding of our study is that while effects of ETC complex I and oxidative phosphorylation inhibition are likely reliant upon reduced ATP/ADP, the effects of complex III inhibition appear to be mediated through ROS production. Scavenging superoxide with tiron or H_2_O_2_ with catalase prevented changes in K_ATP_-dependent lymphatic pacemaker modulation in response to complex III inhibition. It is somewhat surprising that the effects of complex III inhibition could be fully prevented by ROS scavenging as antimycin A also would be expected to have an effect on the ATP/ADP ratio. Nonetheless, modulation of mitochondrial energetics had profound effects on lymphatic contractile function through multiple mechanisms of K_ATP_ activaton.

ATP is a negative regulator of the K_ATP_ channel, with channel activity increasing as ATP levels fall.^30^ But how might ROS activate the K_ATP_ channel? At least two other studies have demonstrated ROS-mediated activation of K_ATP_ channels.^59,60^ Although the exact mechanism is unknown and may vary with the ROS species and/or K_ATP_ subunit composition, one study found that H_2_O_2_ lowers the sensitivity of the channel to ATP, implying that ROS effects are mediated by the SUR channel subunit.^60^ ROS species can also activate protein kinases^61^ and ROS-mediated PKA activation could be another potential mechanism for the increased activity of LMC K_ATP_ channels.^25,62^ However, investigating these mechanisms in lymphatic vessels was beyond the scope of this study.

We have assumed that the K_ATP_ channels controlling lymphatic muscle pacemaking are located on the LMC sarcolemma, based in part on the observation that antimycin A-induced inhibition of ETC inhibited AP generation in LMCs (Figure 4). However, evidence suggests that K_ATP_ channels are also expressed on the mitochondrial inner membrane (mitoKATP), where they regulate ATP production and alter mitochondrial Ca^2+^ uptake by changing the mitochondrial membrane potential.^57^ The molecular identity of mitoKATP has been recently described, MitoK (*Ccdc51*) and MitoSur (*Abcb8*),^63^ but assessment of its role in most intact cell preparations is still limited to the use of pharmacological tools such as the inhibitor 5-hydroxydecanoate and the activator diazoxide. These compounds are often promoted as selective regulators of mitoKATP over sarcolemmal KATP (“sarcKATP”) channels, but their selectivity is controversial^64–67^ and both compounds, or their metabolites, also act on other mitochondrial targets.^67–69^ Given the limitations of these modulators and the intact vessel techniques employed in the present study, we would be unable to definitively discriminate between mitoKATP and sarcKATP in the regulation of lymphatic vessel pacemaking. However, this remains an area for further investigation.

A major implication of our results is that acute metabolic stress leads to the activation of K_ATP_ channels in lymphatic muscle, retarding the ionic pacemaker and inhibiting the spontaneous contractions that are critical for active lymph pumping. Although K_ATP_ channel activity makes little contribution to the pacemaking frequency of murine lymphatic vessels under normoxic conditions,^35,48^ activation of this channel acts as a powerful brake on pacemaking^62^ by inhibiting the repetitive cycle of diastolic depolarization and AP firing involving anoctamin1 and L-type Ca^2+^ channels [for details see recent reviews^62,70^]. Given these findings, we propose that K_ATP_ channel activation is a possible explanation for the impaired lymphatic contractile function and/or lymph transport observed in a number of animal models of metabolic disease. It is interesting that many of these studies, like ours, show an impairment—often a selective impairment—in lymphatic contraction frequency. Several examples are notable. (1) In mice and rats of advanced age, selective reductions in basal lymphatic contraction frequency^71^ and near-elimination of flow-mediated contraction frequency regulation^13^ were noted in comparison to vessels from younger animals. (2) In mice fed a high fat diet (HFD) to induce obesity, a reduced packet frequency of dye transport was observed in lymphatic vessels of the hindlimb.^15,16^ (3) In *ApoE^−/−^* mice on a HFD, contraction frequency and FPF of ex vivo popliteal lymphatics were impaired at low pressures.^6^ (4) In a mouse model of TNFα overexpression, popliteal lymphatic vessels showed a reduced spontaneous contraction frequency over a wide range of intraluminal pressure.^18^ (5) In a high fructose rat model of metabolic syndrome, contraction frequency and calculated pump flow were reduced 40-50%, depending on the intraluminal pressure level, with no significant change in contraction amplitude, stroke volume, or ejection fraction.^21^ (6) In a rat model of experimental ileitis associated with increased prostanoid and nitric oxide production, spontaneous lymphatic contractions were nearly abolished.^23,25^ Although these findings are consistent with the conclusion that K_ATP_ channels mediated the reductions in contraction frequency, only the latter two studies specifically tested their possible role. In the experimental ileitis model, GLIB (10 µm) treatment essentially restored the lymphatic contraction frequency to normal levels,^25^ and in the metabolic syndrome model, GLIB (10 µm) partially restored the depression in contraction frequency.^22^ Collectively, these findings and ours point to a nearly selective impairment of lymphatic contraction frequency in models of metabolic stress, with little or no effect on contraction amplitude, suggesting that a common endpoint of the metabolic stress associated with these various disease models is primarily to inhibit the ionic pacemaker driving spontaneous contractions as opposed to interfering with excitation-contraction coupling. The lack of effect of mitochondrial ETC inhibitors on contraction amplitude in our study suggests that they do not have effects on L-type Ca^2+^ channels, although the slight blunting of contraction amplitude observed during rescue by GLIB may be related to the documented off-target effect of GLIB on the L-type channel.^72–74^ It will be important to test the extent to which GLIB treatment [at lower concentrations that are more specific for K_ATP_ channels in lymphatic muscle^48^] can restore impaired lymphatic function in other animal models of metabolic disease and if animals with lymphatic muscle deficient in Kir6.1 channels are resistant to the lymphatic contractile dysfunction associated with those models.

The preceding discussion is not meant to imply that K_ATP_ channels are the sole cause of lymphatic dysfunction in metabolic disease or to downplay the importance of other signaling mechanisms that might also negatively impact lymphatic contractile amplitude. Indeed, two of the studies cited above^6,18^ noted impairments in lymphatic contraction amplitude as well as frequency. Related studies have demonstrated this as well. For example, Liao et al.^20^ found that upregulation of nitric oxide (NO) production through iNOS (by injecting activated macrophages or CD11b + Gr-1 + cells expressing iNOS) led to the suppression of lymph transport in the mouse hindlimb. The effect appeared to be mediated both by changes in contraction amplitude and frequency, although (as with other in vivo models) a component of the frequency changes may have been due to systemic compensation in order to maintain fluid balance. In these and other studies, the effects on amplitude are likely mediated in part by nitric oxide (NO). For example, in a TNFα overexpression mouse model, the impairment in both contraction frequency and amplitude of ex vivo lymphatic vessels was partially rescued by blocking NO synthase.^18^ Likewise, iNOS inhibition with 1400 W largely restored the lymphatic contractions lost during experimental ileitis.^25^ Inhibition of eNOS by L-NAME partially reversed the impairment in lymphatic contraction frequency and amplitude in a mouse model of TNFα overexpression,^18^ a rat model of aging^71^ and a rat model of metabolic syndrome.^22^ The effects of NO on lymphatic contractile function are complex, but in general, low levels of NO production associated with shear-stress activation of eNOS result in slight enhancement of lymph pump output^75,76^ whereas higher levels of NO production, often associated with imposed flow or iNOS activation, generally depress both lymphatic contraction frequency and amplitude.^18,22,48,62,71^ To complicate matters, NO appears to induce K_ATP_ channel activation in some species^25^ but not others,^48^ perhaps depending on the relative activities of PKA and PKG in lymphatic muscle [for more detail see^62^]. Thus, the extent to which K_ATP_ channels may be activated directly by metabolic stress or secondary to NO and/or prostanoid production is likely to vary with the disease model.

In the context of metabolic disease, lymphatic contractile dysfunction can potentially trigger a complicated sequelae of long-term changes in overall lymphatic function and the interstitium. Such changes include peri-lymphatic inflammation, CD4+ cell infiltration, lipid accumulation, and fibrosis—all of which are associated with ROS production, iNOS activation, and/or the production of cytokines and prostanoids.^24^,^77–80^ For example, in addition to an impairment in lymphatic contractile function,^15,16^ obesity-prone mice show reduced lymphatic transport of macromolecules draining to lymph nodes along with decreased density of lymphatic vessels and reduced expression of lymphatic markers, all suggestive of both short- and long-term remodeling of the lymphatic vasculature and surrounding tissue.^17^ The long-term changes in lymphatic function may negatively impact the lymphatic endothelium, leading to collecting vessel hyperpermeability^6,28,29^ and compromised integrity of lymphatic valves.^6,26,27^ These, together with inhibition of the active lymph pump through K_ATP_ channel activation and/or NO production would contribute to overall lymphatic system dysfunction.

In summary, acute metabolic stress, either through reductions in the intracellular ATP/ADP ratio or increased ROS production, leads to activation of K_ATP_ channels in lymphatic muscle and inhibition of the ionic pacemaker driving spontaneous lymphatic contractions and active lymph transport. Mice lacking functional K_ATP_ channels in lymphatic muscle cells are resistant to the effects of acute metabolic stress, pointing to a common role for K_ATP_ channels in the impaired lymphatic contractile function observed in a number of metabolic diseases and raising the possibility that K_ATP_ channels in lymphatic muscle may be a viable therapeutic target.

## Supplementary Material

zqae033_Supplemental_Files

## Data Availability

All data needed to evaluate the conclusions in the paper are present in the paper.
