# Supplementary figures and images for: Acute Metabolic Stress Induces Lymphatic Dysfunction Through KATP Channel Activation
Source: Function (Oxf). 2024 Jul 29;5(5):zqae033. doi: 10.1093/function/zqae033 (PMC11384908; doi:10.1093/function/zqae033)

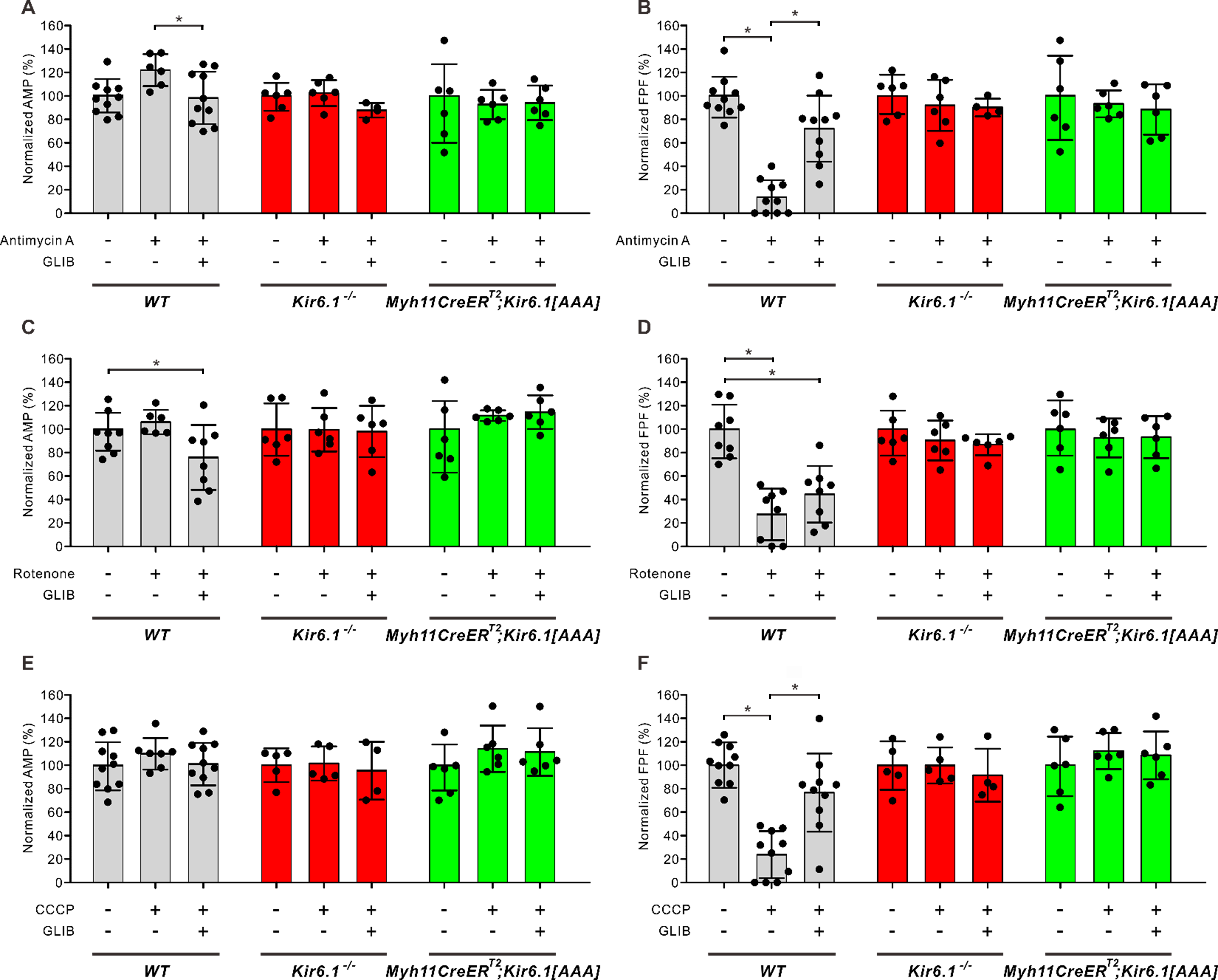

Supplement: zqae033_Supplemental_Files [file zqae033_supplemental_files.zip › Supp Fig1 w bars(450dpi).png]

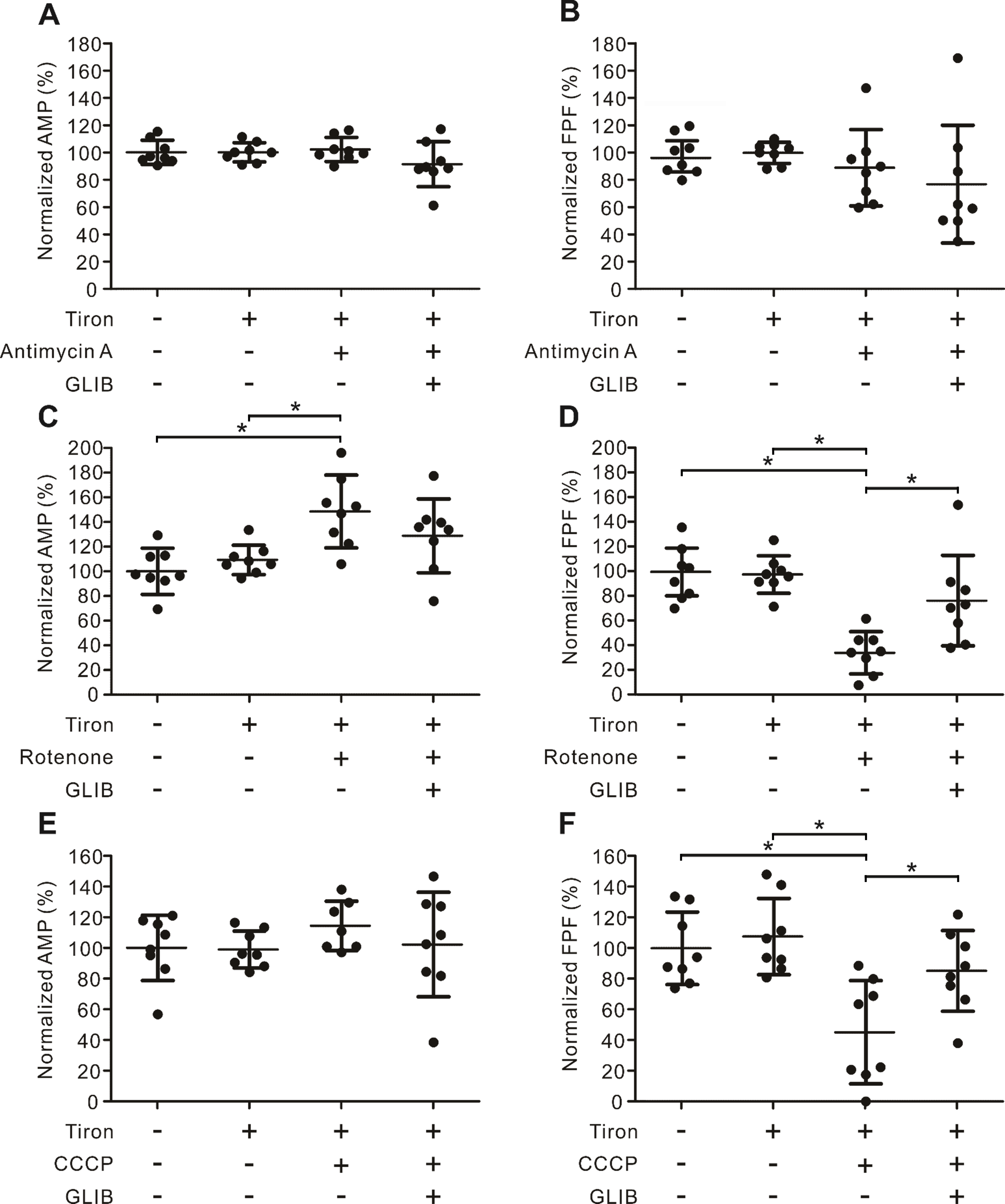

Supplement: zqae033_Supplemental_Files [file zqae033_supplemental_files.zip › Suppl Fig2 w bars(450dpi).png]

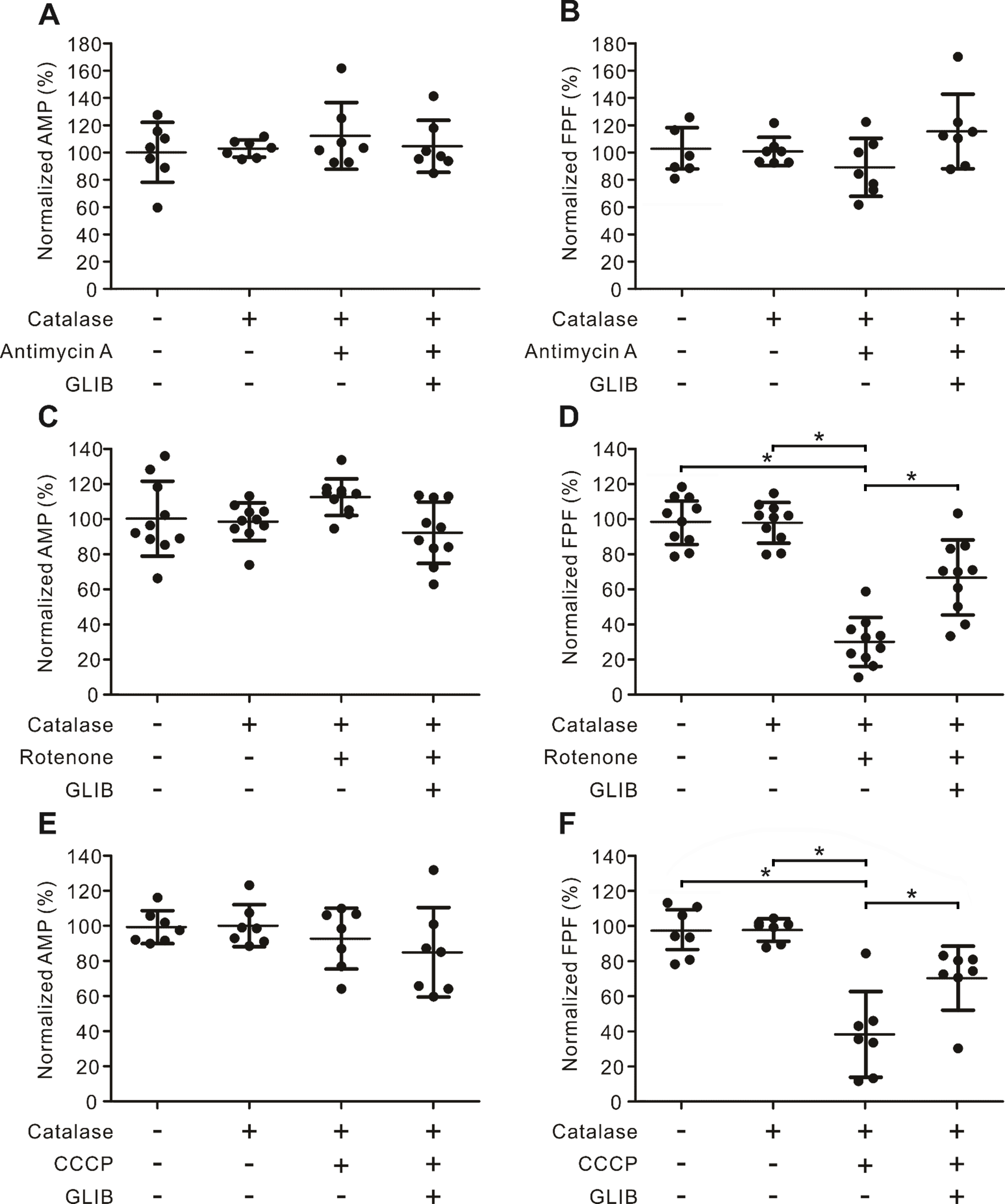

Supplement: zqae033_Supplemental_Files [file zqae033_supplemental_files.zip › Suppl Fig3 w bars(450dpi).png]
